# Crystal structure of (4,4′-bipyridyl-κ*N*)bis­[*N*-(2-hydroxy­ethyl)-*N*-iso­propyl­dithio­carbamato-κ^2^ *S*,*S*′]zinc(II)–4,4′-bipyridyl (2/1) and its isostructural cadmium(II) analogue

**DOI:** 10.1107/S2056989017014396

**Published:** 2017-10-13

**Authors:** Yee Seng Tan, Edward R. T. Tiekink

**Affiliations:** a Research Centre for Crystalline Materials, School of Science and Technology, Sunway University, 47500 Bandar Sunway, Selangor Darul Ehsan, Malaysia

**Keywords:** crystal structure, zinc, cadmium, di­thio­carbamate, 4,4′-bi­pyridine, hydrogen bonding

## Abstract

The title structures, [*M*(C_6_H_12_NOS_2_)_2_(C_10_H_8_N_2_)]·0.5C_10_H_8_N_2_, for *M* = Zn, (I), and Cd, (II), feature terminally bound 4,4′-bipyridyl ligands and non-coordinating 4,4′-bi­pyridyl mol­ecules, with the latter disposed about a centre of inversion. The coordination geometry about the metal atom is defined by two non-symmetrically chelating di­thio­carbamate ligands and a pyridyl N atom. The NS_4_ donor sets are distorted but, approximate to trigonal bipyramidal in each case. In the crystal, hy­droxy-O—H⋯O(hy­droxy) and hy­droxy-O—H⋯N(pyrid­yl) hydrogen bonds between the zinc-containing mol­ecules lead to a supra­molecular layer parallel to (100). The three-dimensional architecture arises as the layers are linked *via* methine-C—H⋯S, pyridyl-C—H⋯O(hy­droxy) and π–π [inter-centroid distance between coordinated pyridyl rings = 3.6246 (18) Å] inter­actions. Channels along the *c*-axis direction are occupied by the non-coordinating 4,4′-bipyridine mol­ecules, which are held in place by C—H⋯π(chelate ring) contacts.

## Abstract 2

The NS_4_ donor set in Zn[S_2_CN(*i*-Pr)CH_2_CH_2_OH]_2_(4,4′-bipyrid­yl).0.5(4,4′-bipyrid­yl), which features monodentate and non-coordinating 4,4′-bipyridyl mol­ecules, is based on a trigonal bipyramid; the cadmium(II) analogue is isostructural.

## Chemical context   

The ditopic ligand 4,4′-bi­pyridyl is ubiquitous in coordination chemistry, usually providing bridges between metal centres to generate coordination polymers. While bidentate bridging is normally observed in the structural chemistry of zinc(II) bis­(*N*,*N*′-di­alkyl­dithio­carbamate)s, these more often than not lead to binuclear species of the general formula [Zn(S_2_CN*RR*′)_2_]_2_(4,4′-bipyrid­yl) as first observed in the archetypal compound [Zn(S_2_CNEt_2_)_2_]_2_(4,4′-bipyrid­yl) (Zem­skova *et al.*, 1994 ▸) and in other compounds relevant to the present study, such as {Zn[S_2_CN(*R*)CH_2_CH_2_OH]_2_}_2_(4,4′-bipyrid­yl) for *R* = Me, Et and CH_2_CH_2_OH (Benson *et al.*, 2007 ▸). The exceptional structure is that of Zn[S_2_CN(*n*-Pr)_2_]_2_(4,4′-bipyrid­yl), which features a relatively rare monodentate coordination mode for the 4,4′-bipyridyl mol­ecule (Klevtsova *et al.*, 2001 ▸). The analogous chemistry for cadmium(II) bis(*N*,*N*′-di­alkyl­dithio­carbamate)s is considerably less explored with the only example in the Cambridge Structural Database (Groom *et al.*, 2016 ▸) being a linear coordination polymer in the crystal of {Cd[S_2_CN(CH_2_Ph)_2_]_2_(4,4′-bipyrid­yl)}_*n*_ (Fan *et al.*, 2007 ▸). The difference in chemistry between zinc and cadmium di­thio­carbamates can be rationalized in terms of the larger size of cadmium *versus* zinc but, also in terms of the reduced Lewis acidity of the zinc atom owing to the strong chelation mode of the di­thio­carbamate ligand. This is also true for cadmium whereby unusual coordination modes are found for related pyridyl-containing mol­ecules that might otherwise be expected to be bridging. This is discussed further below in *Database survey*. In the present report, the crystal and mol­ec­ular structures of two compounds, formulated as Zn[S_2_CN(*i*-Pr)CH_2_CH_2_OH]_2_(4,4′-bipyrid­yl)·0.5(4,4′-bipyrid­yl) (I) and the cadmium analogue (II), are described, *i.e*. featuring monodentate and non-coordinating 4,4′-bi­pyridine mol­ecules.
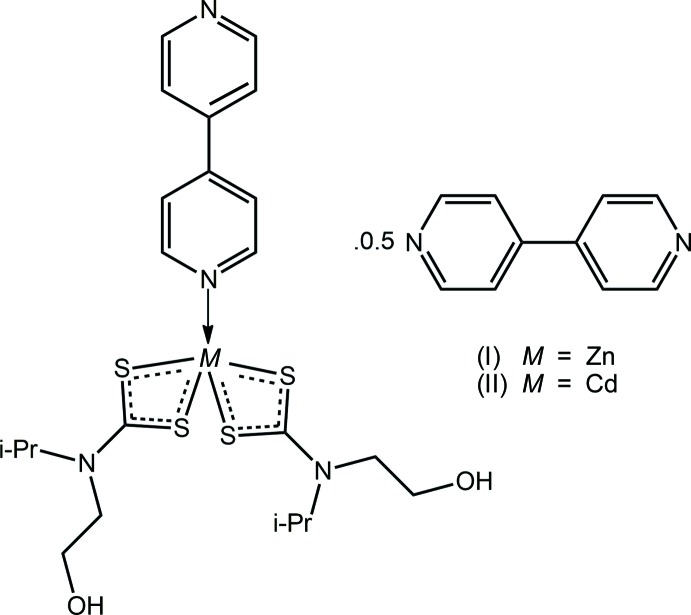


## Structural commentary   

**Figure 1:**
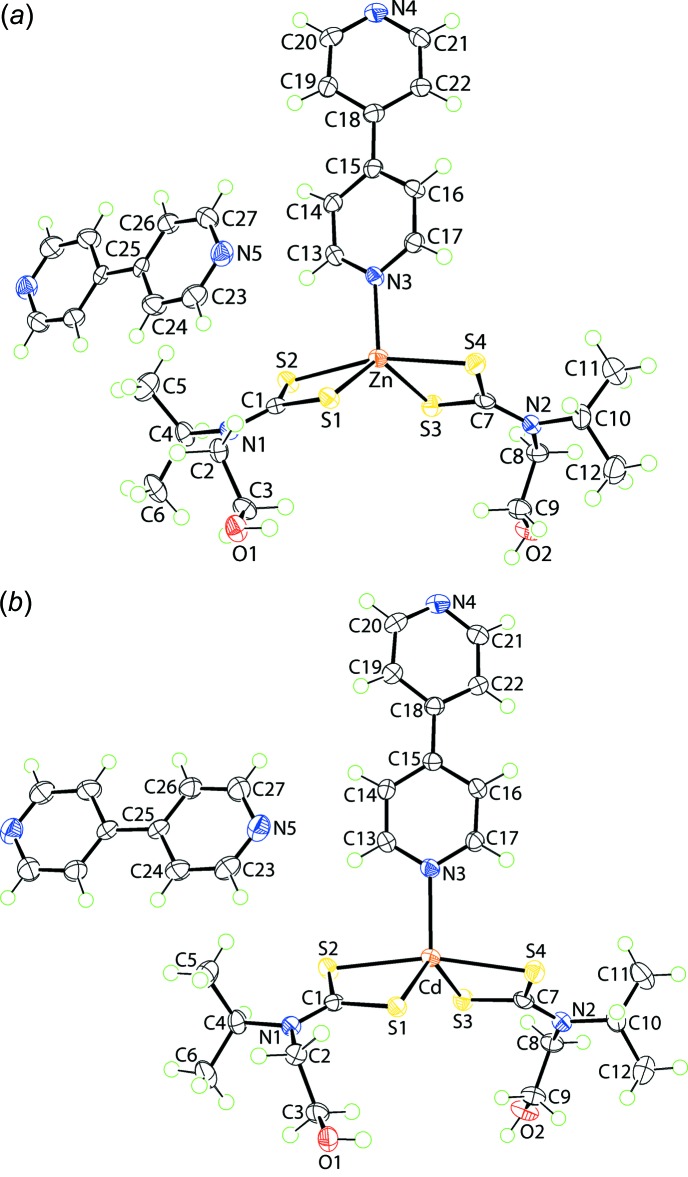
The mol­ecular structures of the constituents of (*a*) (I) and (*b*) (II) showing the atom-labelling scheme and displacement ellipsoids at the 70% probability level. For each of (I) and (II), the 4,4′-bi­pyridine mol­ecule has been expanded to show the entire mol­ecule; unlabelled atoms are related by the symmetry operation −*x*, 2 − *y*, −*z*.

The mol­ecular structure of the constituents of (I) are shown in Fig. 1 ▸ *a* and selected geometric parameters are collected in Table 1 ▸. The asymmetric unit comprises an entire mol­ecule of Zn[S_2_CN(*i*-Pr)CH_2_CH_2_OH]_2_(4,4′-bipyrid­yl) and half a mol­ecule of 4,4′-bi­pyridine, the latter being disposed about a centre of inversion. The zinc atom is coordinated by two di­thio­carbamate ligands that form disparate Zn—S bond lengths. This is seen in the values of Δ(Zn—S) = Zn—S_long_ − Zn—S_short_, which compute to 0.19 and 0.23 Å for the S1- and S3-di­thio­carbamate ligands, respectively. The fifth position in the coordination geometry is occupied by a pyridyl-N atom. Based on the value of τ (Addison *et al.*, 1984 ▸), which equals to 0.0 and 1.0 for ideal square-pyramidal and trigonal–bipyramidal geometries, respectively, it is possible to assign a coordination geometry based on the NS_4_ donor set. In (I), τ = 0.64 indicating a highly distorted coordination geometry but, one approximating a trigonal bipyramid. In this description, the less tightly bound S2 and S4 atoms define the axial positions, Table 1 ▸. The coordinated 4,4′-bipyridyl mol­ecule is non-planar with the dihedral angle between the two residues being 28.12 (14)°.

Crystals of (II) are isostructural to those of (I), Fig. 1 ▸ *b* and Table 2 ▸. Some differences in mol­ecular geometry are apparent, most notably in the degree of symmetry in the Cd—S bond lengths, *i.e*. Δ(Cd—S) = 0.09 and 0.11 Å for the S1- and S3-di­thio­carbamate ligands, respectively. This is reflected in the narrower ranges in the C—S bond lengths in (II) *cf*. (I), Tables 1 ▸ and 2 ▸. The value of τ = 0.67 suggests a coordination geometry marginally closer to trigonal bipyramidal in (II) than for (I). The dihedral angle between the two rings comprising the coordinated 4,4′-bipyridyl mol­ecule is 28.86 (7)°.

## Supra­molecular features   

**Figure 2:**
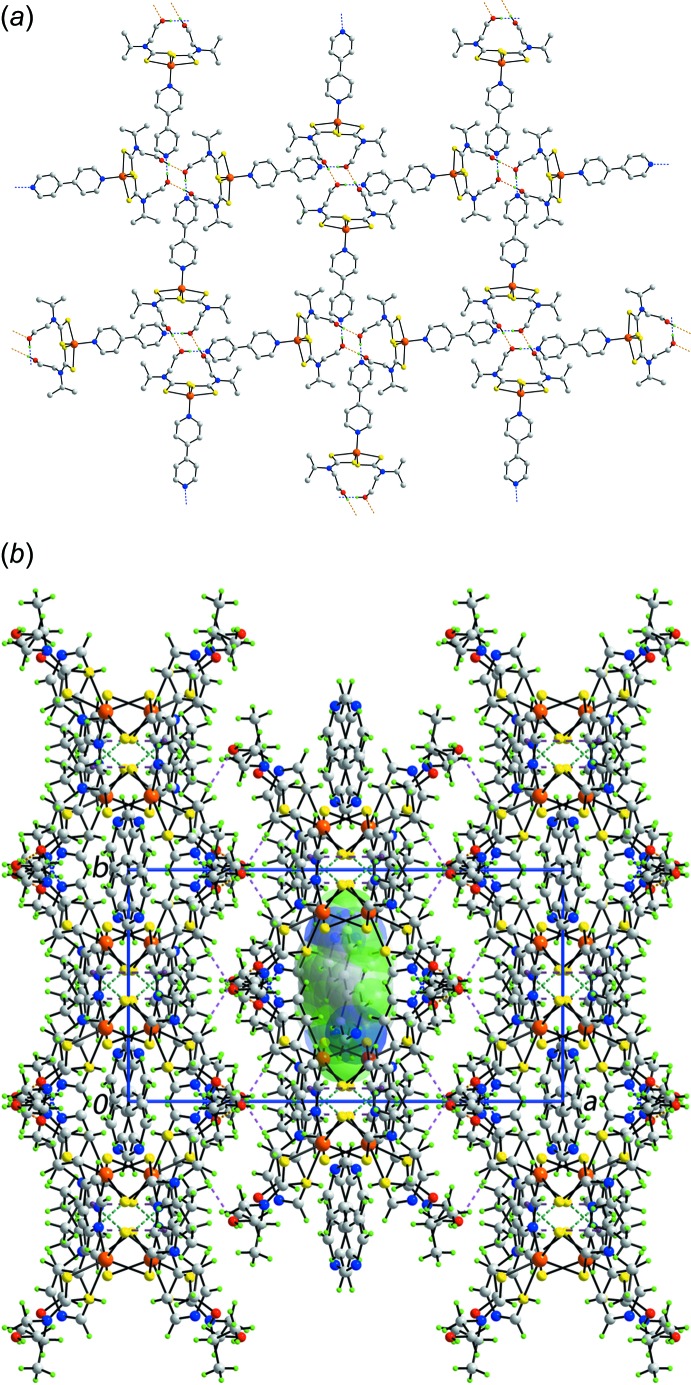
Mol­ecular packing in (I): (*a*) view of two-dimensional supra­molecular array sustained by hy­droxy-O—H⋯O(hy­droxy) and hy­droxy-O–H⋯N(pyrid­yl) hydrogen bonding with all but the acidic H atoms removed and (*b*) a view of the unit-cell contents in projection down the *c* axis, with the non-coordinating 4,4′-bipyridine mol­ecules in one channel highlighted in space-filling mode. The O—H⋯O, O—H⋯N, C—H⋯O, C—H⋯S and π–π inter­actions are shown as orange, blue, pink, sea-blue and purple dashed lines, respectively.

The mol­ecular packing of (I) comprises conventional hydrogen bonding as well as a number of weaker, non-covalent inter­actions, Table 3 ▸. The presence of hy­droxy-O—H⋯O(hy­droxy) hydrogen bonds leads to the formation of a centrosymmetric, 28-membered {⋯HOC_2_NCSZnSCNC_2_O}_2_ synthon. This ring contains two additional hy­droxy-O—H H atoms and these form hy­droxy-O—H⋯N(pyrid­yl) hydrogen bonds with the non-coordinating end of the monodentate 4,4′-bipyridyl mol­ecules. This network of hydrogen bonds leads to the formation of a two-dimensional array lying parallel to (100), Fig. 2 ▸ *a*. These layers are connected into double-layers *via* methine-C—H⋯S and π–π inter­actions involving the coordinated pyridyl ring [inter-centroid distance between the (N3/C13–C17) and (N3/C13–C17)^i^ rings = 3.6246 (18) Å and angle of inclination = 0.46 (13)° for symmetry code (i): −*x*, *y*, 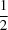 − *z*]. The double-layers are connected into a three-dimensional architecture *via* 4,4′-bipyridyl-C—H⋯O(hy­droxy) inter­actions, involving an H atom from the non-coordinating ring of the coordinated 4,4′-bipyridyl mol­ecule. This architecture defines channels parallel to the *c* axis in which residue the non-coordinating 4,4′-bi­pyridine mol­ecules. The closest inter­action between the host and guests are of the type pyridine-C—H⋯π(Zn/S3/S4/C7), *i.e*. C—H⋯π(chelate ring), a supra­molecular synthon gaining prominence in the structural chemistry of metal-containing species (Tiekink, 2017 ▸), especially for di­thio­carbamates (Tiekink & Zukerman-Schpector, 2011 ▸) owing to the ability of the di­thio­carbamate ligand to form strong chelating inter­actions (see above).

The mol­ecular packing for isostructural (II) follows that just described for (I), Table 4 ▸. However, in this case, the putative pyridyl-C—H⋯π(Cd/S3/S4/C7) inter­action is just beyond the sum of the van der Waals radii for this type of contact (Spek, 2009 ▸).

## Database survey   

As mentioned in the *Chemical context*, ditopic ligands such as 4,4′-bipyridyl are normally observed providing bridges between metal centres. Thus, the structures of (I) and (II) are doubly curious as not only is the 4,4′-bipyridyl ligand coordinating in a monodentate fashion, there is a non-coordinating 4,4′-bi­pyridine mol­ecule in the crystal. Recent reports confirm these inter­esting observations with related bipyridyl-type mol­ecules of both the zinc(II) and, especially, cadmium(II) di­thio­carbamates. Thus, just as for the 4,4′-bipyridyl structures mentioned in the *Chemical context*, *i.e*. [Zn(S_2_CNEt_2_)_2_]_2_(4,4′-bipyrid­yl) (Zemskova *et al.*, 1994 ▸) and Zn[S_2_CN(*n*-Pr)_2_]_2_(4,4′-bipyrid­yl) (Klevtsova *et al.*, 2001 ▸), with the anti­cipated bidentate, bridging and non-anti­cipated terminal coordination, respectively, similar chemistry occurs for the ditopic ligand with an ethyl­ene space, *i.e. trans*-bis­(4-pyrid­yl)ethyl­ene (bpe) where structures of both bridging, *i.e*. [Zn(S_2_CNEt_2_)_2_]_2_(bpe) (Arman *et al.*, 2009 ▸), and terminal, *i.e*. Zn[S_2_CN(*n*-Pr)_2_]_2_(bpe) (Lai & Tiekink, 2003 ▸), coordination modes are known. Very recently, terminal coordination was found for 4-pyridine­aldazine in the structure of Zn[S_2_CN(Me)CH_2_CH_2_OH_2_]_2_(4-pyridine­aldazine) (Broker *et al.*, 2017 ▸). In the realm of cadmium di­thio­carbamates, the potentially bridging ligand just mentioned occurs in the structure of Cd[S_2_CN(*n*-Pr)CH_2_CH_2_OH_2_]_2_(4-pyridine­alda­zine)_2_ with both being terminally bound (Broker & Tiekink, 2011 ▸). The ditopic ligand bpe was mentioned above. In the case of cadmium di­thio­carbamates, a bidentate, bridging mode is seen in the crystal of [Cd(S_2_CNEt_2_)_2_(bpe)]_*n*_ (Chai *et al.*, 2003 ▸). However, in another example both bridging and terminal modes, in a 1:2 ratio, are seen in the structure of Cd[S_2_CN(*i*-Pr)CH_2_CH_2_OH_2_]_2_(bpe)_3_ (Jotani *et al.*, 2016 ▸). The occurrence of unusual coordination modes for these bipyridyl-type ligands indicate additional factors are coming into play, often a competition between hydrogen bonding and *M*←N donor inter­actions but, not always as seen in the structure of Zn[S_2_CN(*n*-Pr)_2_]_2_(4,4′-bipyrid­yl) (Klevtsova *et al.*, 2001 ▸).

## Synthesis and crystallization   

All chemicals and solvents were used as purchased without purification·The melting point was determined using an Krüss KSP1N melting point meter. The IR spectra were obtained by the attenuated total reflectance (ATR) technique on a Perkin Elmer RX1 FTIR spectrophotometer from 4000 to 400 cm^−1. 1^H and ^13^C NMR spectra were recorded at room temperature in DMSO-*d*_6_ solution on a Bruker Avance 400MHz NMR spectrometer.

Synthesis of (I): 4,4′-bi­pyridine (1.79 mmol, 0.28 g) in ethanol (25 ml) was added dropwise to bis­(*N*-2-hy­droxy­ethyl,*N*-iso­propyl­dithio­carbamato)zinc(II) (1.21 mmol, 0.51 g) in ethanol (25 ml). The resulting mixture was stirred for 0.5 h follow by filtration. After a week of slow evaporation of the filtrate, yellow blocks precipitated (yield: 0.698 g, 88%; m.p. 445.6 K). IR (cm^−1^): 1467 (*m*) [ν(C—N)], 1175 (*m*) [ν(C—S)] cm^−1. 1^H NMR: δ 8.78–7.83 (*m*, 12H, aromatic H), 5.14 (*sept*, 2H, NCH, 6.63 Hz), 4.90 (*t*, 2H, OH, 5.38 Hz), 3.78–3.64 (*m*, 8H, NCH_2_CH_2_O), 1.18 (*d*, 12H, CH_3_, 6.72 Hz). ^13^C NMR: δ 204.15 (CS_2_), 150.53, 144.65, 121.58 (aromatic-C), 58.21 (CH_2_O), 55.53 (NCH_2_), 49.80 (NCH), 19.88 (CH_3_).

Synthesis of (II): 4,4′-bi­pyridine (1.61 mmol, 0.25 g) in ethanol (25 ml) was added dropwise to bis­(*N*-2-hy­droxy­ethyl,*N*-iso­propyl­dithio­carbamato)cadmium(II) (1.07 mmol, 0.50 g) in ethanol (25 ml). The resulting mixture was stirred for 0.5 h follow by filtration. A week of slow evaporation of the filtrate yielded yellow blocks (yield: 0.652 g, 87%; m.p. 438.7 K). IR (cm^−1^): 1467 (*m*) [ν(C—N)], 1174 (*m*) [ν(C—S)] cm^−1. 1^H NMR: δ 8.79–7.80 (*m*, 12H, aromatic H), 5.22 (*sept*, 2H, NCH, 6.63 Hz), 4.84 (*t*, 2H, OH, 5.52 Hz), 3.80–3.64 (*m*, 8H, NCH_2_CH_2_O), 1.17 (*d*, 12H, CH_3_, 6.72 Hz). ^13^C NMR: δ 205.29 (CS_2_), 150.57, 144.46, 121.41 (aromatic-C), 58.26 (CH_2_O), 56.62 (NCH_2_), 50.47 (NCH), 19.91 (CH_3_).

## Refinement   

Crystal data, data collection and structure refinement details are summarized in Table 5 ▸. For each of (I) and (II), carbon-bound H atoms were placed in calculated positions (C—H = 0.95–1.00 Å) and were included in the refinement in the riding-model approximation, with *U*_iso_(H) set to 1.2–1.5*U*_eq_(C). The O-bound H atoms were located in difference-Fourier maps but were refined with a distance restraint of O—H = 0.84±0.01 Å, and with *U*_iso_(H) set to 1.5*U*_eq_(O). For (I), owing to poor agreement, two reflections, *i.e*. (0 0 6) and (27 3 4), were omitted from the final cycles of refinement. For (II), one reflection, *i.e*. (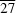 7 7), was omitted for the same reason.

## Supplementary Material

Crystal structure: contains datablock(s) I, II, global. DOI: 10.1107/S2056989017014396/hb7712sup1.cif

Structure factors: contains datablock(s) I. DOI: 10.1107/S2056989017014396/hb7712Isup2.hkl

Structure factors: contains datablock(s) II. DOI: 10.1107/S2056989017014396/hb7712IIsup3.hkl

CCDC references: 1578328, 1578327

Additional supporting information:  crystallographic information; 3D view; checkCIF report

## Figures and Tables

**Table 1:** Selected geometric parameters (Å, °) for (I)

Zn—N3	2.077 (2)	C1—S1	1.733 (3)
Zn—S1	2.3540 (10)	C1—S2	1.715 (3)
Zn—S2	2.5366 (9)	C7—S3	1.735 (3)
Zn—S3	2.3541 (9)	C7—S4	1.714 (3)
Zn—S4	2.5904 (9)		
			
S1—Zn—S3	124.19 (3)	S2—Zn—S4	162.87 (3)

**Table 2:** Selected geometric parameters (Å, °) for (II)

Cd—N3	2.3011 (11)	C1—S1	1.7310 (12)
Cd—S1	2.5547 (3)	C1—S2	1.7218 (12)
Cd—S2	2.6500 (3)	C7—S3	1.7328 (13)
Cd—S3	2.5620 (4)	C7—S4	1.7257 (13)
Cd—S4	2.6696 (4)		
			
S1—Cd—S3	125.725 (11)	S2—Cd—S4	165.865 (11)

**Table 3:** Hydrogen-bond geometry (Å, °) for (II) *Cg*1 is the centroid of the Zn/S3/S4/C7 chelate ring.

*D*—H⋯*A*	*D*—H	H⋯*A*	*D*⋯*A*	*D*—H⋯*A*
O1—H1*O*⋯N4^i^	0.83 (2)	1.88 (2)	2.7085 (15)	176 (1)
O2—H2*O*⋯O1^ii^	0.83 (1)	1.89 (1)	2.7162 (14)	173 (2)
C4—H4⋯S2^iii^	1.00	2.68	3.5395 (14)	144
C22—H22⋯O2^iv^	0.95	2.40	3.3473 (17)	174
C26—H26⋯*Cg*1^v^	0.95	3.00	3.776 (3)	140

Symmetry codes: (i) 
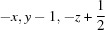
; (ii) 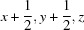; (iii) 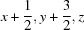; (iv) 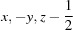; (v) 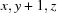.

**Table 4:** Hydrogen-bond geometry (Å, °) for (I) *Cg*1 is the centroid of the Cd/S3/S4/C7 chelate ring.

*D*—H⋯*A*	*D*—H	H⋯*A*	*D*⋯*A*	*D*—H⋯*A*
O1—H1*O*⋯N4^i^	0.85 (2)	1.85 (2)	2.697 (3)	179 (3)
O2—H2*O*⋯O1^ii^	0.84 (2)	1.88 (2)	2.718 (3)	176 (4)
C4—H4⋯S2^iii^	1.00	2.67	3.515 (3)	142
C22—H22⋯O2^iv^	0.95	2.36	3.300 (3)	170
C26—H26⋯*Cg*1^v^	0.95	3.04	3.7943 (15)	138

Symmetry codes: (i) 
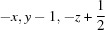
; (ii) 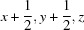; (iii) 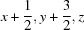; (iv) 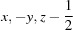; (v) 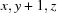.

**Table 5:** Experimental details

	(I)	(II)
Crystal data		
Chemical formula	[Zn(C_6_H_12_NOS_2_)_2_(C_10_H_8_N_2_)]·0.5C_10_H_8_N_2_	[Cd(C_6_H_12_NOS_2_)_2_(C_10_H_8_N_2_)]·0.5C_10_H_8_N_2_
*M*_r_	656.22	703.25
Crystal system, space group	Monoclinic, *C*2/*c*	Monoclinic, *C*2/*c*
Temperature (K)	100	100
*a*, *b*, *c* (Å)	22.418 (5), 11.501 (2), 25.094 (5)	22.7028 (12), 11.5950 (6), 24.8196 (13)
β (°)	105.50 (3)	103.385 (1)
*V* (Å^3^)	6235 (2)	6356.0 (6)
*Z*	8	8
Radiation type	Mo *K*α	Mo *K*α
μ (mm^−1^)	1.09	0.98
Crystal size (mm)	0.30 × 0.20 × 0.20	0.04 × 0.04 × 0.03

Data collection		
Diffractometer	Bruker SMART APEX CCD	Bruker SMART APEX CCD
Absorption correction	Multi-scan (*SADABS*; Sheldrick, 1996 ▸)	Multi-scan (*SADABS*; Sheldrick, 1996 ▸)
*T*_min_, *T*_max_	0.968, 0.979	0.962, 0.971
No. of measured, independent and observed [*I* > 2σ(*I*)] reflections	31204, 7738, 5204	41284, 7847, 7204
*R*_int_	0.082	0.023
(sin θ/λ)_max_ (Å^−1^)	0.667	0.667

Refinement		
*R*[*F* ^2^ > 2σ(*F* ^2^)], *wR*(*F* ^2^), *S*	0.041, 0.096, 1.00	0.019, 0.049, 0.99
No. of reflections	7738	7847
No. of parameters	358	358
No. of restraints	2	2
H-atom treatment	H atoms treated by a mixture of independent and constrained refinement	H atoms treated by a mixture of independent and constrained refinement
Δρ_max_, Δρ_min_ (e Å^−3^)	0.48, −0.46	0.46, −0.24

Computer programs: *APEX2* and *SAINT* (Bruker, 2008 ▸), *SHELXS97* (Sheldrick, 2008 ▸), *SHELXL2014* (Sheldrick, 2015 ▸), *ORTEP-3 for Windows* (Farrugia, 2012 ▸), *DIAMOND* (Brandenburg, 2006 ▸) and *publCIF* (Westrip, 2010 ▸).

## supplementary crystallographic information

### (4,4'-Bipyridyl-κN)bis[N-(2-hydroxyethyl)-N-isopropyldithiocarbamato-κ2S,S']zinc(II)–4,4'-bipyridyl (2/1) (I) Crystal data

**Table:**

[Zn(C_6_H_12_NOS_2_)_2_(C_10_H_8_N_2_)]·0.5C_10_H_8_N_2_	*F*(000) = 2744
*M**_r_* = 656.22	*D*_x_ = 1.398 Mg m^−^^3^
Monoclinic, *C*2/*c*	Mo *K*α radiation, λ = 0.71073 Å
*a* = 22.418 (5) Å	Cell parameters from 3045 reflections
*b* = 11.501 (2) Å	θ = 2.3–25.2°
*c* = 25.094 (5) Å	µ = 1.09 mm^−^^1^
β = 105.50 (3)°	*T* = 100 K
*V* = 6235 (2) Å^3^	Block, yellow
*Z* = 8	0.30 × 0.20 × 0.20 mm

### (4,4'-Bipyridyl-κN)bis[N-(2-hydroxyethyl)-N-isopropyldithiocarbamato-κ2S,S']zinc(II)–4,4'-bipyridyl (2/1) (I) Data collection

**Table:**

Bruker SMART APEX CCD diffractometer	7738 independent reflections
Radiation source: fine-focus sealed tube	5204 reflections with *I* > 2σ(*I*)
Graphite monochromator	*R*_int_ = 0.082
ω scans	θ_max_ = 28.3°, θ_min_ = 2.0°
Absorption correction: multi-scan (SADABS; Sheldrick, 1996)	*h* = −27→29
*T*_min_ = 0.968, *T*_max_ = 0.979	*k* = −15→15
31204 measured reflections	*l* = −33→33

### (4,4'-Bipyridyl-κN)bis[N-(2-hydroxyethyl)-N-isopropyldithiocarbamato-κ2S,S']zinc(II)–4,4'-bipyridyl (2/1) (I) Refinement

**Table:**

Refinement on *F*^2^	Primary atom site location: structure-invariant direct methods
Least-squares matrix: full	Hydrogen site location: mixed
*R*[*F*^2^ > 2σ(*F*^2^)] = 0.041	H atoms treated by a mixture of independent and constrained refinement
*wR*(*F*^2^) = 0.096	*w* = 1/[σ^2^(*F*_o_^2^) + (0.0351*P*)^2^] where *P* = (*F*_o_^2^ + 2*F*_c_^2^)/3
*S* = 1.00	(Δ/σ)_max_ < 0.001
7738 reflections	Δρ_max_ = 0.48 e Å^−^^3^
358 parameters	Δρ_min_ = −0.46 e Å^−^^3^
2 restraints	

### (4,4'-Bipyridyl-κN)bis[N-(2-hydroxyethyl)-N-isopropyldithiocarbamato-κ2S,S']zinc(II)–4,4'-bipyridyl (2/1) (I) Special details

**Table:**

Geometry. All esds (except the esd in the dihedral angle between two l.s. planes) are estimated using the full covariance matrix. The cell esds are taken into account individually in the estimation of esds in distances, angles and torsion angles; correlations between esds in cell parameters are only used when they are defined by crystal symmetry. An approximate (isotropic) treatment of cell esds is used for estimating esds involving l.s. planes.

### (4,4'-Bipyridyl-κN)bis[N-(2-hydroxyethyl)-N-isopropyldithiocarbamato-κ2S,S']zinc(II)–4,4'-bipyridyl (2/1) (I) Fractional atomic coordinates and isotropic or equivalent isotropic displacement parameters (Å^2^)

**Table:**

	*x*	*y*	*z*	*U*_iso_*/*U*_eq_	
Zn	0.05280 (2)	0.31725 (3)	0.16518 (2)	0.01632 (9)	
S1	−0.04838 (3)	0.24709 (6)	0.12760 (3)	0.01626 (15)	
S2	0.00828 (3)	0.43350 (6)	0.07767 (3)	0.01723 (15)	
S3	0.14139 (3)	0.24004 (6)	0.14471 (3)	0.01887 (16)	
S4	0.09136 (3)	0.14774 (6)	0.23371 (3)	0.01927 (16)	
O1	−0.20135 (9)	0.08393 (16)	0.01233 (8)	0.0200 (4)	
H1O	−0.1869 (13)	0.034 (2)	0.0372 (9)	0.030*	
O2	0.25910 (9)	−0.01819 (18)	0.09286 (8)	0.0250 (5)	
H2O	0.2425 (14)	−0.036 (3)	0.0599 (6)	0.038*	
N1	−0.10592 (10)	0.35160 (18)	0.03253 (9)	0.0138 (5)	
N2	0.18772 (10)	0.05318 (19)	0.20497 (9)	0.0162 (5)	
N3	0.06894 (10)	0.45571 (18)	0.22034 (9)	0.0147 (5)	
N4	0.15344 (12)	0.9270 (2)	0.40786 (10)	0.0240 (6)	
N5	0.02413 (12)	0.7932 (2)	0.11006 (10)	0.0260 (6)	
C1	−0.05450 (12)	0.3457 (2)	0.07419 (11)	0.0140 (5)	
C2	−0.15890 (12)	0.2760 (2)	0.03229 (11)	0.0157 (6)	
H2A	−0.1974	0.3119	0.0093	0.019*	
H2B	−0.1630	0.2682	0.0704	0.019*	
C3	−0.15066 (13)	0.1563 (2)	0.00952 (11)	0.0180 (6)	
H3A	−0.1488	0.1632	−0.0293	0.022*	
H3B	−0.1114	0.1214	0.0314	0.022*	
C4	−0.11249 (13)	0.4398 (2)	−0.01199 (11)	0.0183 (6)	
H4	−0.0699	0.4635	−0.0129	0.022*	
C5	−0.14485 (14)	0.5472 (2)	0.00182 (13)	0.0280 (7)	
H5A	−0.1490	0.6049	−0.0277	0.042*	
H5B	−0.1860	0.5258	0.0052	0.042*	
H5C	−0.1204	0.5802	0.0369	0.042*	
C6	−0.14513 (14)	0.3914 (3)	−0.06898 (11)	0.0265 (7)	
H6A	−0.1484	0.4524	−0.0969	0.040*	
H6B	−0.1213	0.3259	−0.0776	0.040*	
H6C	−0.1867	0.3647	−0.0691	0.040*	
C7	0.14472 (13)	0.1375 (2)	0.19613 (11)	0.0167 (6)	
C8	0.23385 (12)	0.0495 (2)	0.17274 (11)	0.0185 (6)	
H8A	0.2719	0.0110	0.1950	0.022*	
H8B	0.2448	0.1300	0.1650	0.022*	
C9	0.21025 (13)	−0.0148 (3)	0.11867 (11)	0.0208 (6)	
H9A	0.1976	−0.0947	0.1255	0.025*	
H9B	0.1740	0.0259	0.0947	0.025*	
C10	0.19577 (13)	−0.0287 (2)	0.25202 (11)	0.0216 (6)	
H10	0.1560	−0.0303	0.2629	0.026*	
C11	0.24603 (15)	0.0160 (3)	0.30141 (12)	0.0342 (8)	
H11A	0.2511	−0.0384	0.3324	0.051*	
H11B	0.2852	0.0227	0.2912	0.051*	
H11C	0.2341	0.0925	0.3124	0.051*	
C12	0.20898 (15)	−0.1525 (2)	0.23619 (13)	0.0294 (7)	
H12A	0.2140	−0.2037	0.2683	0.044*	
H12B	0.1744	−0.1801	0.2061	0.044*	
H12C	0.2471	−0.1532	0.2241	0.044*	
C13	0.06136 (13)	0.5664 (2)	0.20303 (11)	0.0190 (6)	
H13	0.0458	0.5809	0.1645	0.023*	
C14	0.07504 (12)	0.6604 (2)	0.23849 (11)	0.0182 (6)	
H14	0.0692	0.7373	0.2242	0.022*	
C15	0.09742 (12)	0.6424 (2)	0.29521 (11)	0.0158 (6)	
C16	0.10366 (12)	0.5272 (2)	0.31327 (11)	0.0165 (6)	
H16	0.1175	0.5102	0.3517	0.020*	
C17	0.08975 (12)	0.4380 (2)	0.27525 (11)	0.0169 (6)	
H17	0.0951	0.3602	0.2884	0.020*	
C18	0.11560 (13)	0.7408 (2)	0.33461 (11)	0.0172 (6)	
C19	0.08885 (13)	0.8507 (2)	0.32338 (12)	0.0208 (6)	
H19	0.0571	0.8641	0.2903	0.025*	
C20	0.10898 (14)	0.9398 (2)	0.36078 (12)	0.0235 (7)	
H20	0.0902	1.0141	0.3526	0.028*	
C21	0.17857 (14)	0.8211 (2)	0.41830 (12)	0.0251 (7)	
H21	0.2101	0.8102	0.4517	0.030*	
C22	0.16164 (13)	0.7269 (2)	0.38367 (11)	0.0192 (6)	
H22	0.1811	0.6536	0.3932	0.023*	
C23	−0.00233 (15)	0.7643 (2)	0.05770 (13)	0.0277 (7)	
H23	−0.0148	0.6858	0.0501	0.033*	
C24	−0.01294 (14)	0.8414 (2)	0.01340 (12)	0.0256 (7)	
H24	−0.0318	0.8151	−0.0231	0.031*	
C25	0.00438 (12)	0.9574 (2)	0.02306 (11)	0.0152 (6)	
C26	0.03155 (13)	0.9879 (2)	0.07766 (11)	0.0218 (6)	
H26	0.0444	1.0658	0.0868	0.026*	
C27	0.03987 (14)	0.9051 (3)	0.11860 (12)	0.0260 (7)	
H27	0.0581	0.9292	0.1556	0.031*	

### (4,4'-Bipyridyl-κN)bis[N-(2-hydroxyethyl)-N-isopropyldithiocarbamato-κ2S,S']zinc(II)–4,4'-bipyridyl (2/1) (I) Atomic displacement parameters (Å^2^)

**Table:**

	*U*^11^	*U*^22^	*U*^33^	*U*^12^	*U*^13^	*U*^23^
Zn	0.01770 (18)	0.01443 (16)	0.01542 (16)	−0.00168 (14)	0.00199 (13)	−0.00196 (13)
S1	0.0195 (4)	0.0144 (3)	0.0137 (3)	−0.0037 (3)	0.0024 (3)	0.0018 (3)
S2	0.0190 (4)	0.0162 (3)	0.0149 (3)	−0.0062 (3)	0.0019 (3)	0.0018 (3)
S3	0.0212 (4)	0.0169 (3)	0.0191 (4)	−0.0009 (3)	0.0062 (3)	0.0018 (3)
S4	0.0232 (4)	0.0183 (3)	0.0172 (4)	0.0032 (3)	0.0071 (3)	0.0019 (3)
O1	0.0201 (11)	0.0164 (10)	0.0207 (11)	−0.0046 (8)	0.0006 (9)	0.0059 (8)
O2	0.0193 (11)	0.0349 (12)	0.0216 (11)	−0.0052 (9)	0.0066 (9)	−0.0113 (10)
N1	0.0157 (12)	0.0126 (11)	0.0126 (11)	−0.0023 (9)	0.0031 (9)	0.0012 (8)
N2	0.0159 (12)	0.0178 (12)	0.0139 (11)	0.0007 (10)	0.0020 (9)	−0.0006 (9)
N3	0.0134 (12)	0.0159 (11)	0.0134 (11)	−0.0017 (9)	0.0010 (9)	−0.0001 (9)
N4	0.0313 (15)	0.0186 (12)	0.0204 (13)	0.0010 (11)	0.0039 (11)	−0.0049 (10)
N5	0.0279 (15)	0.0205 (13)	0.0284 (14)	0.0004 (11)	0.0055 (12)	0.0037 (11)
C1	0.0161 (14)	0.0120 (12)	0.0146 (13)	−0.0001 (11)	0.0050 (11)	−0.0028 (10)
C2	0.0113 (14)	0.0175 (13)	0.0176 (14)	−0.0014 (11)	0.0025 (11)	0.0039 (11)
C3	0.0165 (15)	0.0202 (14)	0.0163 (14)	−0.0059 (11)	0.0026 (11)	−0.0010 (11)
C4	0.0195 (15)	0.0186 (14)	0.0150 (14)	−0.0025 (12)	0.0014 (11)	0.0064 (11)
C5	0.0345 (19)	0.0170 (15)	0.0354 (18)	0.0029 (13)	0.0142 (15)	0.0076 (13)
C6	0.0271 (18)	0.0318 (17)	0.0162 (15)	−0.0034 (14)	−0.0020 (13)	0.0073 (13)
C7	0.0175 (15)	0.0154 (13)	0.0147 (14)	−0.0044 (11)	0.0003 (11)	−0.0047 (10)
C8	0.0153 (15)	0.0213 (14)	0.0189 (14)	−0.0005 (12)	0.0044 (12)	−0.0045 (11)
C9	0.0166 (15)	0.0242 (15)	0.0216 (15)	−0.0037 (12)	0.0047 (12)	−0.0049 (12)
C10	0.0215 (16)	0.0227 (15)	0.0200 (15)	0.0053 (13)	0.0046 (12)	0.0054 (12)
C11	0.039 (2)	0.0365 (19)	0.0228 (17)	0.0076 (16)	0.0007 (15)	0.0012 (14)
C12	0.0344 (19)	0.0234 (16)	0.0299 (18)	0.0057 (14)	0.0076 (15)	0.0068 (13)
C13	0.0201 (15)	0.0198 (14)	0.0143 (14)	0.0016 (12)	−0.0002 (12)	0.0007 (11)
C14	0.0198 (15)	0.0136 (13)	0.0195 (14)	0.0014 (11)	0.0022 (12)	−0.0002 (11)
C15	0.0115 (14)	0.0179 (14)	0.0181 (14)	0.0011 (11)	0.0042 (11)	−0.0021 (11)
C16	0.0193 (15)	0.0183 (14)	0.0115 (13)	−0.0017 (12)	0.0035 (11)	−0.0010 (11)
C17	0.0191 (15)	0.0138 (13)	0.0179 (14)	−0.0003 (11)	0.0050 (12)	0.0010 (11)
C18	0.0200 (16)	0.0154 (13)	0.0172 (14)	−0.0008 (11)	0.0067 (12)	−0.0008 (11)
C19	0.0212 (16)	0.0180 (14)	0.0206 (15)	0.0032 (12)	0.0012 (12)	−0.0015 (11)
C20	0.0289 (17)	0.0160 (14)	0.0250 (16)	0.0019 (13)	0.0065 (13)	−0.0040 (12)
C21	0.0324 (18)	0.0223 (15)	0.0161 (14)	0.0017 (14)	−0.0013 (13)	−0.0025 (12)
C22	0.0239 (16)	0.0163 (14)	0.0166 (14)	0.0008 (12)	0.0042 (12)	−0.0023 (11)
C23	0.0342 (19)	0.0154 (14)	0.0330 (18)	−0.0033 (13)	0.0077 (15)	−0.0007 (13)
C24	0.0342 (19)	0.0179 (15)	0.0233 (16)	−0.0031 (13)	0.0051 (14)	−0.0013 (12)
C25	0.0120 (14)	0.0135 (13)	0.0203 (14)	0.0026 (11)	0.0049 (11)	0.0014 (11)
C26	0.0270 (17)	0.0109 (13)	0.0255 (16)	−0.0030 (12)	0.0035 (13)	−0.0013 (11)
C27	0.0295 (18)	0.0244 (16)	0.0208 (16)	−0.0003 (14)	0.0009 (13)	−0.0022 (13)

### (4,4'-Bipyridyl-κN)bis[N-(2-hydroxyethyl)-N-isopropyldithiocarbamato-κ2S,S']zinc(II)–4,4'-bipyridyl (2/1) (I) Geometric parameters (Å, º)

**Table:**

Zn—N3	2.077 (2)	C8—H8A	0.9900
Zn—S1	2.3540 (10)	C8—H8B	0.9900
Zn—S2	2.5366 (9)	C9—H9A	0.9900
Zn—S3	2.3541 (9)	C9—H9B	0.9900
Zn—S4	2.5904 (9)	C10—C11	1.525 (4)
C1—S1	1.733 (3)	C10—C12	1.529 (4)
C1—S2	1.715 (3)	C10—H10	1.0000
C7—S3	1.735 (3)	C11—H11A	0.9800
C7—S4	1.714 (3)	C11—H11B	0.9800
O1—C3	1.426 (3)	C11—H11C	0.9800
O1—H1O	0.843 (10)	C12—H12A	0.9800
O2—C9	1.414 (3)	C12—H12B	0.9800
O2—H2O	0.837 (10)	C12—H12C	0.9800
N1—C1	1.335 (3)	C13—C14	1.381 (4)
N1—C2	1.471 (3)	C13—H13	0.9500
N1—C4	1.486 (3)	C14—C15	1.392 (4)
N2—C7	1.343 (3)	C14—H14	0.9500
N2—C8	1.474 (3)	C15—C16	1.396 (4)
N2—C10	1.483 (3)	C15—C18	1.486 (4)
N3—C13	1.341 (3)	C16—C17	1.378 (4)
N3—C17	1.347 (3)	C16—H16	0.9500
N4—C20	1.335 (4)	C17—H17	0.9500
N4—C21	1.338 (3)	C18—C22	1.388 (4)
N5—C23	1.331 (4)	C18—C19	1.395 (4)
N5—C27	1.337 (4)	C19—C20	1.381 (4)
C2—C3	1.520 (4)	C19—H19	0.9500
C2—H2A	0.9900	C20—H20	0.9500
C2—H2B	0.9900	C21—C22	1.377 (4)
C3—H3A	0.9900	C21—H21	0.9500
C3—H3B	0.9900	C22—H22	0.9500
C4—C5	1.519 (4)	C23—C24	1.391 (4)
C4—C6	1.527 (4)	C23—H23	0.9500
C4—H4	1.0000	C24—C25	1.393 (4)
C5—H5A	0.9800	C24—H24	0.9500
C5—H5B	0.9800	C25—C26	1.388 (4)
C5—H5C	0.9800	C25—C25^i^	1.489 (5)
C6—H6A	0.9800	C26—C27	1.377 (4)
C6—H6B	0.9800	C26—H26	0.9500
C6—H6C	0.9800	C27—H27	0.9500
C8—C9	1.512 (4)		
			
N3—Zn—S1	120.49 (7)	O2—C9—C8	107.2 (2)
N3—Zn—S3	115.32 (7)	O2—C9—H9A	110.3
S1—Zn—S3	124.19 (3)	C8—C9—H9A	110.3
N3—Zn—S2	97.53 (6)	O2—C9—H9B	110.3
S1—Zn—S2	73.73 (3)	C8—C9—H9B	110.3
S3—Zn—S2	99.76 (3)	H9A—C9—H9B	108.5
N3—Zn—S4	99.60 (6)	N2—C10—C11	109.8 (2)
S1—Zn—S4	97.06 (3)	N2—C10—C12	112.1 (2)
S3—Zn—S4	73.12 (3)	C11—C10—C12	111.9 (2)
S2—Zn—S4	162.87 (3)	N2—C10—H10	107.6
C1—S1—Zn	87.38 (9)	C11—C10—H10	107.6
C1—S2—Zn	82.03 (9)	C12—C10—H10	107.6
C7—S3—Zn	88.01 (10)	C10—C11—H11A	109.5
C7—S4—Zn	81.07 (10)	C10—C11—H11B	109.5
C3—O1—H1O	106 (2)	H11A—C11—H11B	109.5
C9—O2—H2O	105 (2)	C10—C11—H11C	109.5
C1—N1—C2	120.0 (2)	H11A—C11—H11C	109.5
C1—N1—C4	121.0 (2)	H11B—C11—H11C	109.5
C2—N1—C4	118.8 (2)	C10—C12—H12A	109.5
C7—N2—C8	120.5 (2)	C10—C12—H12B	109.5
C7—N2—C10	121.3 (2)	H12A—C12—H12B	109.5
C8—N2—C10	117.7 (2)	C10—C12—H12C	109.5
C13—N3—C17	117.0 (2)	H12A—C12—H12C	109.5
C13—N3—Zn	121.84 (18)	H12B—C12—H12C	109.5
C17—N3—Zn	121.11 (17)	N3—C13—C14	123.2 (2)
C20—N4—C21	116.7 (2)	N3—C13—H13	118.4
C23—N5—C27	115.3 (3)	C14—C13—H13	118.4
N1—C1—S2	122.3 (2)	C13—C14—C15	120.0 (2)
N1—C1—S1	120.8 (2)	C13—C14—H14	120.0
S2—C1—S1	116.84 (15)	C15—C14—H14	120.0
N1—C2—C3	111.0 (2)	C14—C15—C16	116.8 (2)
N1—C2—H2A	109.4	C14—C15—C18	121.8 (2)
C3—C2—H2A	109.4	C16—C15—C18	121.4 (2)
N1—C2—H2B	109.4	C17—C16—C15	119.8 (2)
C3—C2—H2B	109.4	C17—C16—H16	120.1
H2A—C2—H2B	108.0	C15—C16—H16	120.1
O1—C3—C2	109.4 (2)	N3—C17—C16	123.2 (2)
O1—C3—H3A	109.8	N3—C17—H17	118.4
C2—C3—H3A	109.8	C16—C17—H17	118.4
O1—C3—H3B	109.8	C22—C18—C19	117.4 (2)
C2—C3—H3B	109.8	C22—C18—C15	120.6 (2)
H3A—C3—H3B	108.2	C19—C18—C15	121.9 (2)
N1—C4—C5	109.9 (2)	C20—C19—C18	119.4 (3)
N1—C4—C6	112.4 (2)	C20—C19—H19	120.3
C5—C4—C6	111.8 (2)	C18—C19—H19	120.3
N1—C4—H4	107.5	N4—C20—C19	123.4 (3)
C5—C4—H4	107.5	N4—C20—H20	118.3
C6—C4—H4	107.5	C19—C20—H20	118.3
C4—C5—H5A	109.5	N4—C21—C22	124.2 (3)
C4—C5—H5B	109.5	N4—C21—H21	117.9
H5A—C5—H5B	109.5	C22—C21—H21	117.9
C4—C5—H5C	109.5	C21—C22—C18	118.9 (3)
H5A—C5—H5C	109.5	C21—C22—H22	120.6
H5B—C5—H5C	109.5	C18—C22—H22	120.6
C4—C6—H6A	109.5	N5—C23—C24	124.5 (3)
C4—C6—H6B	109.5	N5—C23—H23	117.8
H6A—C6—H6B	109.5	C24—C23—H23	117.8
C4—C6—H6C	109.5	C23—C24—C25	119.4 (3)
H6A—C6—H6C	109.5	C23—C24—H24	120.3
H6B—C6—H6C	109.5	C25—C24—H24	120.3
N2—C7—S4	122.3 (2)	C26—C25—C24	116.3 (2)
N2—C7—S3	120.0 (2)	C26—C25—C25^i^	122.2 (3)
S4—C7—S3	117.69 (16)	C24—C25—C25^i^	121.5 (3)
N2—C8—C9	112.2 (2)	C27—C26—C25	119.9 (3)
N2—C8—H8A	109.2	C27—C26—H26	120.1
C9—C8—H8A	109.2	C25—C26—H26	120.1
N2—C8—H8B	109.2	N5—C27—C26	124.7 (3)
C9—C8—H8B	109.2	N5—C27—H27	117.7
H8A—C8—H8B	107.9	C26—C27—H27	117.7
			
C2—N1—C1—S2	−178.61 (19)	C17—N3—C13—C14	1.4 (4)
C4—N1—C1—S2	−3.1 (3)	Zn—N3—C13—C14	−176.2 (2)
C2—N1—C1—S1	1.6 (3)	N3—C13—C14—C15	−0.5 (4)
C4—N1—C1—S1	177.10 (19)	C13—C14—C15—C16	−1.3 (4)
Zn—S2—C1—N1	−178.6 (2)	C13—C14—C15—C18	177.0 (3)
Zn—S2—C1—S1	1.23 (13)	C14—C15—C16—C17	2.1 (4)
Zn—S1—C1—N1	178.5 (2)	C18—C15—C16—C17	−176.2 (3)
Zn—S1—C1—S2	−1.31 (14)	C13—N3—C17—C16	−0.5 (4)
C1—N1—C2—C3	−83.1 (3)	Zn—N3—C17—C16	177.1 (2)
C4—N1—C2—C3	101.3 (3)	C15—C16—C17—N3	−1.2 (4)
N1—C2—C3—O1	177.3 (2)	C14—C15—C18—C22	−150.2 (3)
C1—N1—C4—C5	−93.7 (3)	C16—C15—C18—C22	28.0 (4)
C2—N1—C4—C5	81.8 (3)	C14—C15—C18—C19	27.9 (4)
C1—N1—C4—C6	141.0 (3)	C16—C15—C18—C19	−153.9 (3)
C2—N1—C4—C6	−43.5 (3)	C22—C18—C19—C20	0.2 (4)
C8—N2—C7—S4	−178.27 (18)	C15—C18—C19—C20	−178.0 (3)
C10—N2—C7—S4	−6.7 (3)	C21—N4—C20—C19	−0.4 (4)
C8—N2—C7—S3	2.4 (3)	C18—C19—C20—N4	0.2 (5)
C10—N2—C7—S3	173.96 (19)	C20—N4—C21—C22	0.4 (5)
Zn—S4—C7—N2	−176.4 (2)	N4—C21—C22—C18	−0.1 (5)
Zn—S4—C7—S3	2.93 (13)	C19—C18—C22—C21	−0.2 (4)
Zn—S3—C7—N2	176.2 (2)	C15—C18—C22—C21	177.9 (3)
Zn—S3—C7—S4	−3.19 (14)	C27—N5—C23—C24	1.1 (5)
C7—N2—C8—C9	−86.4 (3)	N5—C23—C24—C25	−0.4 (5)
C10—N2—C8—C9	101.7 (3)	C23—C24—C25—C26	−0.1 (4)
N2—C8—C9—O2	−176.9 (2)	C23—C24—C25—C25^i^	178.3 (3)
C7—N2—C10—C11	−94.0 (3)	C24—C25—C26—C27	−0.1 (4)
C8—N2—C10—C11	77.8 (3)	C25^i^—C25—C26—C27	−178.5 (3)
C7—N2—C10—C12	140.9 (3)	C23—N5—C27—C26	−1.3 (5)
C8—N2—C10—C12	−47.3 (3)	C25—C26—C27—N5	0.8 (5)

Symmetry code: (i) −*x*, −*y*+2, −*z*.

### (4,4'-Bipyridyl-κN)bis[N-(2-hydroxyethyl)-N-isopropyldithiocarbamato-κ2S,S']zinc(II)–4,4'-bipyridyl (2/1) (I) Hydrogen-bond geometry (Å, º)

Cg1 is the centroid of the Cd/S3/S4/C7 chelate ring.

**Table:**

*D*—H···*A*	*D*—H	H···*A*	*D*···*A*	*D*—H···*A*
O1—H1*O*···N4^ii^	0.85 (2)	1.85 (2)	2.697 (3)	179 (3)
O2—H2*O*···O1^iii^	0.84 (2)	1.88 (2)	2.718 (3)	176 (4)
C4—H4···S2^iv^	1.00	2.67	3.515 (3)	142
C22—H22···O2^v^	0.95	2.36	3.300 (3)	170
C26—H26···*Cg*1^vi^	0.95	3.04	3.7943 (15)	138

Symmetry codes: (ii) −*x*, *y*−1, −*z*+1/2; (iii) *x*+1/2, *y*+1/2, *z*; (iv) *x*+1/2, *y*+3/2, *z*; (v) *x*, −*y*, *z*−1/2; (vi) *x*, *y*+1, *z*.

### (4,4'-Bipyridyl-κN)bis(N-2-hydroxyethyl-N-isopropyldithiocarbamato-κ2S,S')cadmium(II)–4,4'-bipyridyl (2/1) (II) Crystal data

**Table:**

[Cd(C_6_H_12_NOS_2_)_2_(C_10_H_8_N_2_)]·0.5C_10_H_8_N_2_	*F*(000) = 2888
*M**_r_* = 703.25	*D*_x_ = 1.470 Mg m^−^^3^
Monoclinic, *C*2/*c*	Mo *K*α radiation, λ = 0.71073 Å
*a* = 22.7028 (12) Å	Cell parameters from 24754 reflections
*b* = 11.5950 (6) Å	θ = 2.2–28.3°
*c* = 24.8196 (13) Å	µ = 0.98 mm^−^^1^
β = 103.385 (1)°	*T* = 100 K
*V* = 6356.0 (6) Å^3^	Block, yellow
*Z* = 8	0.04 × 0.04 × 0.03 mm

### (4,4'-Bipyridyl-κN)bis(N-2-hydroxyethyl-N-isopropyldithiocarbamato-κ2S,S')cadmium(II)–4,4'-bipyridyl (2/1) (II) Data collection

**Table:**

Bruker SMART APEX CCD diffractometer	7847 independent reflections
Radiation source: fine-focus sealed tube	7204 reflections with *I* > 2σ(*I*)
Graphite monochromator	*R*_int_ = 0.023
ω scans	θ_max_ = 28.3°, θ_min_ = 1.7°
Absorption correction: multi-scan (SADABS; Sheldrick, 1996)	*h* = −30→30
*T*_min_ = 0.962, *T*_max_ = 0.971	*k* = −15→15
41284 measured reflections	*l* = −33→33

### (4,4'-Bipyridyl-κN)bis(N-2-hydroxyethyl-N-isopropyldithiocarbamato-κ2S,S')cadmium(II)–4,4'-bipyridyl (2/1) (II) Refinement

**Table:**

Refinement on *F*^2^	Primary atom site location: structure-invariant direct methods
Least-squares matrix: full	Hydrogen site location: mixed
*R*[*F*^2^ > 2σ(*F*^2^)] = 0.019	H atoms treated by a mixture of independent and constrained refinement
*wR*(*F*^2^) = 0.049	*w* = 1/[σ^2^(*F*_o_^2^) + (0.0249*P*)^2^ + 6.291*P*] where *P* = (*F*_o_^2^ + 2*F*_c_^2^)/3
*S* = 0.99	(Δ/σ)_max_ = 0.001
7847 reflections	Δρ_max_ = 0.46 e Å^−^^3^
358 parameters	Δρ_min_ = −0.24 e Å^−^^3^
2 restraints	

### (4,4'-Bipyridyl-κN)bis(N-2-hydroxyethyl-N-isopropyldithiocarbamato-κ2S,S')cadmium(II)–4,4'-bipyridyl (2/1) (II) Special details

**Table:**

Geometry. All esds (except the esd in the dihedral angle between two l.s. planes) are estimated using the full covariance matrix. The cell esds are taken into account individually in the estimation of esds in distances, angles and torsion angles; correlations between esds in cell parameters are only used when they are defined by crystal symmetry. An approximate (isotropic) treatment of cell esds is used for estimating esds involving l.s. planes.

### (4,4'-Bipyridyl-κN)bis(N-2-hydroxyethyl-N-isopropyldithiocarbamato-κ2S,S')cadmium(II)–4,4'-bipyridyl (2/1) (II) Fractional atomic coordinates and isotropic or equivalent isotropic displacement parameters (Å^2^)

**Table:**

	*x*	*y*	*z*	*U*_iso_*/*U*_eq_	
Cd	0.04996 (2)	0.31263 (2)	0.16486 (2)	0.01723 (3)	
S1	−0.05744 (2)	0.24286 (3)	0.12217 (2)	0.01723 (6)	
S2	0.00085 (2)	0.43727 (3)	0.07636 (2)	0.01907 (7)	
S3	0.14726 (2)	0.23031 (3)	0.14448 (2)	0.02008 (7)	
S4	0.09406 (2)	0.14429 (3)	0.23619 (2)	0.02109 (7)	
O1	−0.20171 (4)	0.08734 (8)	0.00837 (4)	0.02070 (19)	
H1O	−0.1878 (8)	0.0408 (13)	0.0337 (6)	0.031*	
O2	0.26295 (4)	−0.02139 (9)	0.09436 (4)	0.0253 (2)	
H2O	0.2468 (8)	−0.0409 (17)	0.0620 (5)	0.038*	
N1	−0.10858 (5)	0.35363 (9)	0.02905 (4)	0.0151 (2)	
N2	0.19087 (5)	0.05102 (10)	0.20849 (4)	0.0180 (2)	
N3	0.06811 (5)	0.46557 (9)	0.22553 (4)	0.0166 (2)	
N4	0.15540 (6)	0.92852 (10)	0.41323 (5)	0.0257 (2)	
N5	0.02716 (6)	0.79642 (11)	0.11132 (6)	0.0294 (3)	
C1	−0.05999 (5)	0.34592 (10)	0.07123 (5)	0.0146 (2)	
C2	−0.16139 (5)	0.27871 (11)	0.02738 (5)	0.0169 (2)	
H2A	−0.1980	0.3156	0.0045	0.020*	
H2B	−0.1676	0.2693	0.0653	0.020*	
C3	−0.15277 (6)	0.16054 (11)	0.00341 (5)	0.0189 (2)	
H3A	−0.1516	0.1683	−0.0360	0.023*	
H3B	−0.1139	0.1266	0.0237	0.023*	
C4	−0.11285 (6)	0.44310 (11)	−0.01475 (5)	0.0187 (2)	
H4	−0.0706	0.4674	−0.0149	0.022*	
C5	−0.14574 (7)	0.54876 (13)	−0.00028 (7)	0.0298 (3)	
H5A	−0.1484	0.6072	−0.0293	0.045*	
H5B	−0.1866	0.5269	0.0025	0.045*	
H5C	−0.1234	0.5803	0.0352	0.045*	
C6	−0.14156 (7)	0.39715 (14)	−0.07228 (6)	0.0269 (3)	
H6A	−0.1434	0.4589	−0.0996	0.040*	
H6B	−0.1172	0.3332	−0.0811	0.040*	
H6C	−0.1826	0.3698	−0.0732	0.040*	
C7	0.14824 (6)	0.13339 (11)	0.19783 (5)	0.0176 (2)	
C8	0.23758 (6)	0.04441 (12)	0.17638 (5)	0.0198 (3)	
H8A	0.2740	0.0063	0.1990	0.024*	
H8B	0.2491	0.1234	0.1677	0.024*	
C9	0.21556 (6)	−0.02239 (12)	0.12279 (6)	0.0216 (3)	
H9A	0.2055	−0.1027	0.1309	0.026*	
H9B	0.1788	0.0143	0.0999	0.026*	
C10	0.19723 (6)	−0.02799 (12)	0.25659 (6)	0.0235 (3)	
H10	0.1579	−0.0276	0.2682	0.028*	
C11	0.24587 (8)	0.01794 (16)	0.30508 (6)	0.0369 (4)	
H11A	0.2500	−0.0343	0.3368	0.055*	
H11B	0.2846	0.0229	0.2941	0.055*	
H11C	0.2342	0.0948	0.3154	0.055*	
C12	0.20978 (7)	−0.15172 (13)	0.24188 (7)	0.0302 (3)	
H12A	0.2136	−0.2008	0.2747	0.045*	
H12B	0.1763	−0.1796	0.2124	0.045*	
H12C	0.2475	−0.1544	0.2291	0.045*	
C13	0.06012 (6)	0.57559 (11)	0.20834 (5)	0.0184 (2)	
H13	0.0437	0.5903	0.1702	0.022*	
C14	0.07479 (6)	0.66804 (11)	0.24394 (5)	0.0182 (2)	
H14	0.0688	0.7446	0.2301	0.022*	
C15	0.09848 (5)	0.64880 (11)	0.30033 (5)	0.0153 (2)	
C16	0.10526 (6)	0.53450 (11)	0.31809 (5)	0.0168 (2)	
H16	0.1201	0.5173	0.3562	0.020*	
C17	0.09027 (6)	0.44634 (11)	0.27985 (5)	0.0172 (2)	
H17	0.0959	0.3689	0.2925	0.021*	
C18	0.11718 (6)	0.74550 (11)	0.33969 (5)	0.0167 (2)	
C19	0.08995 (6)	0.85379 (12)	0.33087 (6)	0.0226 (3)	
H19	0.0577	0.8671	0.2995	0.027*	
C20	0.11034 (7)	0.94170 (12)	0.36829 (6)	0.0263 (3)	
H20	0.0914	1.0150	0.3617	0.032*	
C21	0.18115 (7)	0.82418 (12)	0.42167 (6)	0.0241 (3)	
H21	0.2131	0.8134	0.4535	0.029*	
C22	0.16396 (6)	0.73140 (11)	0.38674 (5)	0.0196 (2)	
H22	0.1837	0.6590	0.3946	0.024*	
C23	−0.00188 (8)	0.76757 (13)	0.05982 (7)	0.0323 (3)	
H23	−0.0154	0.6902	0.0533	0.039*	
C24	−0.01356 (7)	0.84325 (12)	0.01538 (6)	0.0288 (3)	
H24	−0.0343	0.8172	−0.0203	0.035*	
C25	0.00530 (6)	0.95788 (11)	0.02321 (5)	0.0188 (2)	
C26	0.03523 (7)	0.98828 (12)	0.07680 (6)	0.0258 (3)	
H26	0.0490	1.0651	0.0848	0.031*	
C27	0.04485 (7)	0.90635 (13)	0.11837 (7)	0.0299 (3)	
H27	0.0655	0.9299	0.1545	0.036*	

### (4,4'-Bipyridyl-κN)bis(N-2-hydroxyethyl-N-isopropyldithiocarbamato-κ2S,S')cadmium(II)–4,4'-bipyridyl (2/1) (II) Atomic displacement parameters (Å^2^)

**Table:**

	*U*^11^	*U*^22^	*U*^33^	*U*^12^	*U*^13^	*U*^23^
Cd	0.01892 (5)	0.01605 (5)	0.01519 (5)	−0.00140 (3)	0.00083 (3)	−0.00184 (3)
S1	0.02100 (15)	0.01453 (14)	0.01510 (14)	−0.00512 (11)	0.00201 (11)	0.00145 (11)
S2	0.02016 (15)	0.01946 (15)	0.01593 (14)	−0.00819 (12)	0.00078 (11)	0.00279 (11)
S3	0.02200 (15)	0.02007 (15)	0.01891 (15)	−0.00138 (12)	0.00624 (12)	0.00193 (12)
S4	0.02419 (16)	0.02194 (16)	0.01883 (15)	0.00403 (12)	0.00847 (12)	0.00277 (12)
O1	0.0189 (4)	0.0182 (4)	0.0216 (5)	−0.0049 (4)	−0.0023 (4)	0.0050 (4)
O2	0.0196 (5)	0.0355 (6)	0.0214 (5)	−0.0060 (4)	0.0062 (4)	−0.0109 (4)
N1	0.0155 (5)	0.0150 (5)	0.0151 (5)	−0.0024 (4)	0.0041 (4)	0.0010 (4)
N2	0.0174 (5)	0.0204 (5)	0.0158 (5)	−0.0001 (4)	0.0028 (4)	−0.0006 (4)
N3	0.0167 (5)	0.0170 (5)	0.0150 (5)	−0.0016 (4)	0.0016 (4)	−0.0009 (4)
N4	0.0340 (7)	0.0196 (6)	0.0230 (6)	−0.0005 (5)	0.0057 (5)	−0.0054 (5)
N5	0.0313 (7)	0.0235 (6)	0.0328 (7)	0.0015 (5)	0.0061 (5)	0.0050 (5)
C1	0.0170 (6)	0.0129 (5)	0.0146 (5)	−0.0020 (4)	0.0052 (4)	−0.0016 (4)
C2	0.0135 (5)	0.0172 (6)	0.0198 (6)	−0.0028 (4)	0.0036 (5)	0.0001 (5)
C3	0.0172 (6)	0.0188 (6)	0.0192 (6)	−0.0040 (5)	0.0015 (5)	−0.0023 (5)
C4	0.0186 (6)	0.0207 (6)	0.0167 (6)	−0.0018 (5)	0.0036 (5)	0.0054 (5)
C5	0.0379 (8)	0.0205 (7)	0.0345 (8)	0.0032 (6)	0.0156 (7)	0.0084 (6)
C6	0.0281 (7)	0.0324 (8)	0.0175 (6)	−0.0029 (6)	−0.0002 (5)	0.0056 (6)
C7	0.0188 (6)	0.0182 (6)	0.0146 (5)	−0.0037 (5)	0.0014 (4)	−0.0033 (5)
C8	0.0168 (6)	0.0228 (6)	0.0197 (6)	−0.0019 (5)	0.0038 (5)	−0.0047 (5)
C9	0.0192 (6)	0.0248 (7)	0.0211 (6)	−0.0047 (5)	0.0055 (5)	−0.0067 (5)
C10	0.0236 (7)	0.0269 (7)	0.0191 (6)	0.0044 (5)	0.0031 (5)	0.0046 (5)
C11	0.0408 (9)	0.0442 (10)	0.0199 (7)	0.0038 (8)	−0.0047 (6)	0.0004 (7)
C12	0.0320 (8)	0.0254 (7)	0.0336 (8)	0.0054 (6)	0.0087 (6)	0.0083 (6)
C13	0.0188 (6)	0.0203 (6)	0.0149 (6)	0.0009 (5)	0.0013 (5)	0.0010 (5)
C14	0.0192 (6)	0.0159 (6)	0.0182 (6)	0.0024 (5)	0.0018 (5)	0.0019 (5)
C15	0.0131 (5)	0.0164 (6)	0.0160 (6)	0.0000 (4)	0.0028 (4)	−0.0020 (4)
C16	0.0175 (6)	0.0187 (6)	0.0136 (5)	−0.0006 (5)	0.0026 (4)	0.0011 (4)
C17	0.0184 (6)	0.0158 (6)	0.0166 (6)	−0.0018 (5)	0.0025 (5)	0.0015 (5)
C18	0.0187 (6)	0.0158 (6)	0.0164 (6)	−0.0008 (5)	0.0055 (5)	−0.0009 (5)
C19	0.0254 (7)	0.0199 (6)	0.0213 (6)	0.0050 (5)	0.0032 (5)	−0.0009 (5)
C20	0.0337 (8)	0.0170 (6)	0.0273 (7)	0.0053 (6)	0.0055 (6)	−0.0029 (5)
C21	0.0283 (7)	0.0224 (7)	0.0188 (6)	−0.0006 (5)	−0.0005 (5)	−0.0034 (5)
C22	0.0228 (6)	0.0166 (6)	0.0188 (6)	0.0019 (5)	0.0036 (5)	−0.0012 (5)
C23	0.0436 (9)	0.0152 (6)	0.0365 (8)	−0.0051 (6)	0.0062 (7)	0.0002 (6)
C24	0.0392 (8)	0.0171 (6)	0.0284 (7)	−0.0059 (6)	0.0045 (6)	−0.0032 (6)
C25	0.0169 (6)	0.0146 (6)	0.0256 (7)	0.0011 (5)	0.0067 (5)	−0.0018 (5)
C26	0.0272 (7)	0.0182 (6)	0.0291 (7)	−0.0026 (5)	0.0010 (6)	−0.0026 (5)
C27	0.0328 (8)	0.0253 (7)	0.0284 (7)	−0.0006 (6)	0.0003 (6)	0.0005 (6)

### (4,4'-Bipyridyl-κN)bis(N-2-hydroxyethyl-N-isopropyldithiocarbamato-κ2S,S')cadmium(II)–4,4'-bipyridyl (2/1) (II) Geometric parameters (Å, º)

**Table:**

Cd—N3	2.3011 (11)	C8—H8A	0.9900
Cd—S1	2.5547 (3)	C8—H8B	0.9900
Cd—S2	2.6500 (3)	C9—H9A	0.9900
Cd—S3	2.5620 (4)	C9—H9B	0.9900
Cd—S4	2.6696 (4)	C10—C12	1.523 (2)
C1—S1	1.7310 (12)	C10—C11	1.528 (2)
C1—S2	1.7218 (12)	C10—H10	1.0000
C7—S3	1.7328 (13)	C11—H11A	0.9800
C7—S4	1.7257 (13)	C11—H11B	0.9800
O1—C3	1.4259 (15)	C11—H11C	0.9800
O1—H1O	0.833 (9)	C12—H12A	0.9800
O2—C9	1.4168 (16)	C12—H12B	0.9800
O2—H2O	0.832 (9)	C12—H12C	0.9800
N1—C1	1.3364 (16)	C13—C14	1.3801 (18)
N1—C2	1.4733 (15)	C13—H13	0.9500
N1—C4	1.4895 (16)	C14—C15	1.3960 (17)
N2—C7	1.3416 (17)	C14—H14	0.9500
N2—C8	1.4688 (16)	C15—C16	1.3940 (17)
N2—C10	1.4851 (17)	C15—C18	1.4832 (17)
N3—C13	1.3441 (16)	C16—C17	1.3827 (17)
N3—C17	1.3441 (16)	C16—H16	0.9500
N4—C20	1.3363 (19)	C17—H17	0.9500
N4—C21	1.3389 (18)	C18—C19	1.3942 (18)
N5—C27	1.3356 (19)	C18—C22	1.3944 (18)
N5—C23	1.338 (2)	C19—C20	1.3846 (19)
C2—C3	1.5242 (18)	C19—H19	0.9500
C2—H2A	0.9900	C20—H20	0.9500
C2—H2B	0.9900	C21—C22	1.3801 (18)
C3—H3A	0.9900	C21—H21	0.9500
C3—H3B	0.9900	C22—H22	0.9500
C4—C5	1.520 (2)	C23—C24	1.386 (2)
C4—C6	1.5220 (19)	C23—H23	0.9500
C4—H4	1.0000	C24—C25	1.3960 (18)
C5—H5A	0.9800	C24—H24	0.9500
C5—H5B	0.9800	C25—C26	1.3917 (19)
C5—H5C	0.9800	C25—C25^i^	1.487 (3)
C6—H6A	0.9800	C26—C27	1.382 (2)
C6—H6B	0.9800	C26—H26	0.9500
C6—H6C	0.9800	C27—H27	0.9500
C8—C9	1.5202 (18)		
			
N3—Cd—S1	121.64 (3)	O2—C9—C8	107.32 (10)
N3—Cd—S3	112.61 (3)	O2—C9—H9A	110.3
S1—Cd—S3	125.725 (11)	C8—C9—H9A	110.3
N3—Cd—S2	95.71 (3)	O2—C9—H9B	110.3
S1—Cd—S2	69.571 (10)	C8—C9—H9B	110.3
S3—Cd—S2	104.839 (11)	H9A—C9—H9B	108.5
N3—Cd—S4	98.43 (3)	N2—C10—C12	112.15 (11)
S1—Cd—S4	102.645 (11)	N2—C10—C11	109.64 (12)
S3—Cd—S4	69.533 (10)	C12—C10—C11	112.07 (13)
S2—Cd—S4	165.865 (11)	N2—C10—H10	107.6
C1—S1—Cd	87.22 (4)	C12—C10—H10	107.6
C1—S2—Cd	84.39 (4)	C11—C10—H10	107.6
C7—S3—Cd	87.13 (4)	C10—C11—H11A	109.5
C7—S4—Cd	83.88 (5)	C10—C11—H11B	109.5
C3—O1—H1O	106.5 (13)	H11A—C11—H11B	109.5
C9—O2—H2O	105.2 (13)	C10—C11—H11C	109.5
C1—N1—C2	120.10 (10)	H11A—C11—H11C	109.5
C1—N1—C4	121.37 (10)	H11B—C11—H11C	109.5
C2—N1—C4	118.36 (10)	C10—C12—H12A	109.5
C7—N2—C8	120.56 (11)	C10—C12—H12B	109.5
C7—N2—C10	121.95 (11)	H12A—C12—H12B	109.5
C8—N2—C10	117.13 (11)	C10—C12—H12C	109.5
C13—N3—C17	117.88 (11)	H12A—C12—H12C	109.5
C13—N3—Cd	122.21 (8)	H12B—C12—H12C	109.5
C17—N3—Cd	119.83 (8)	N3—C13—C14	122.62 (12)
C20—N4—C21	117.14 (12)	N3—C13—H13	118.7
C27—N5—C23	115.51 (13)	C14—C13—H13	118.7
N1—C1—S2	121.29 (9)	C13—C14—C15	119.85 (12)
N1—C1—S1	120.01 (9)	C13—C14—H14	120.1
S2—C1—S1	118.69 (7)	C15—C14—H14	120.1
N1—C2—C3	111.34 (10)	C16—C15—C14	117.23 (11)
N1—C2—H2A	109.4	C16—C15—C18	121.11 (11)
C3—C2—H2A	109.4	C14—C15—C18	121.64 (11)
N1—C2—H2B	109.4	C17—C16—C15	119.62 (11)
C3—C2—H2B	109.4	C17—C16—H16	120.2
H2A—C2—H2B	108.0	C15—C16—H16	120.2
O1—C3—C2	109.17 (10)	N3—C17—C16	122.77 (12)
O1—C3—H3A	109.8	N3—C17—H17	118.6
C2—C3—H3A	109.8	C16—C17—H17	118.6
O1—C3—H3B	109.8	C19—C18—C22	117.47 (12)
C2—C3—H3B	109.8	C19—C18—C15	121.90 (12)
H3A—C3—H3B	108.3	C22—C18—C15	120.62 (11)
N1—C4—C5	109.96 (11)	C20—C19—C18	119.37 (13)
N1—C4—C6	112.47 (11)	C20—C19—H19	120.3
C5—C4—C6	112.12 (12)	C18—C19—H19	120.3
N1—C4—H4	107.3	N4—C20—C19	123.24 (13)
C5—C4—H4	107.3	N4—C20—H20	118.4
C6—C4—H4	107.3	C19—C20—H20	118.4
C4—C5—H5A	109.5	N4—C21—C22	123.81 (13)
C4—C5—H5B	109.5	N4—C21—H21	118.1
H5A—C5—H5B	109.5	C22—C21—H21	118.1
C4—C5—H5C	109.5	C21—C22—C18	118.98 (12)
H5A—C5—H5C	109.5	C21—C22—H22	120.5
H5B—C5—H5C	109.5	C18—C22—H22	120.5
C4—C6—H6A	109.5	N5—C23—C24	124.26 (14)
C4—C6—H6B	109.5	N5—C23—H23	117.9
H6A—C6—H6B	109.5	C24—C23—H23	117.9
C4—C6—H6C	109.5	C23—C24—C25	119.73 (14)
H6A—C6—H6C	109.5	C23—C24—H24	120.1
H6B—C6—H6C	109.5	C25—C24—H24	120.1
N2—C7—S4	121.13 (10)	C26—C25—C24	116.13 (13)
N2—C7—S3	119.58 (10)	C26—C25—C25^i^	121.98 (15)
S4—C7—S3	119.30 (8)	C24—C25—C25^i^	121.88 (15)
N2—C8—C9	111.65 (10)	C27—C26—C25	119.81 (13)
N2—C8—H8A	109.3	C27—C26—H26	120.1
C9—C8—H8A	109.3	C25—C26—H26	120.1
N2—C8—H8B	109.3	N5—C27—C26	124.56 (14)
C9—C8—H8B	109.3	N5—C27—H27	117.7
H8A—C8—H8B	108.0	C26—C27—H27	117.7
			
C2—N1—C1—S2	−176.55 (9)	C17—N3—C13—C14	1.25 (19)
C4—N1—C1—S2	−1.29 (16)	Cd—N3—C13—C14	−175.45 (10)
C2—N1—C1—S1	3.28 (16)	N3—C13—C14—C15	−0.6 (2)
C4—N1—C1—S1	178.54 (9)	C13—C14—C15—C16	−1.00 (19)
Cd—S2—C1—N1	−176.80 (10)	C13—C14—C15—C18	177.49 (12)
Cd—S2—C1—S1	3.36 (6)	C14—C15—C16—C17	1.82 (18)
Cd—S1—C1—N1	176.69 (10)	C18—C15—C16—C17	−176.68 (12)
Cd—S1—C1—S2	−3.48 (7)	C13—N3—C17—C16	−0.37 (19)
C1—N1—C2—C3	−83.28 (14)	Cd—N3—C17—C16	176.41 (9)
C4—N1—C2—C3	101.32 (13)	C15—C16—C17—N3	−1.19 (19)
N1—C2—C3—O1	173.57 (10)	C16—C15—C18—C19	−152.60 (13)
C1—N1—C4—C5	−93.77 (14)	C14—C15—C18—C19	28.96 (19)
C2—N1—C4—C5	81.57 (14)	C16—C15—C18—C22	28.66 (18)
C1—N1—C4—C6	140.52 (12)	C14—C15—C18—C22	−149.77 (13)
C2—N1—C4—C6	−44.15 (15)	C22—C18—C19—C20	0.4 (2)
C8—N2—C7—S4	−178.55 (9)	C15—C18—C19—C20	−178.33 (13)
C10—N2—C7—S4	−5.59 (17)	C21—N4—C20—C19	−0.3 (2)
C8—N2—C7—S3	1.70 (16)	C18—C19—C20—N4	−0.1 (2)
C10—N2—C7—S3	174.65 (10)	C20—N4—C21—C22	0.3 (2)
Cd—S4—C7—N2	−176.04 (10)	N4—C21—C22—C18	0.1 (2)
Cd—S4—C7—S3	3.71 (7)	C19—C18—C22—C21	−0.45 (19)
Cd—S3—C7—N2	175.91 (10)	C15—C18—C22—C21	178.35 (12)
Cd—S3—C7—S4	−3.85 (7)	C27—N5—C23—C24	0.4 (2)
C7—N2—C8—C9	−84.72 (15)	N5—C23—C24—C25	−0.2 (3)
C10—N2—C8—C9	102.00 (13)	C23—C24—C25—C26	−0.1 (2)
N2—C8—C9—O2	178.55 (11)	C23—C24—C25—C25^i^	179.10 (16)
C7—N2—C10—C12	138.65 (13)	C24—C25—C26—C27	0.3 (2)
C8—N2—C10—C12	−48.17 (16)	C25^i^—C25—C26—C27	−178.94 (16)
C7—N2—C10—C11	−96.19 (15)	C23—N5—C27—C26	−0.2 (2)
C8—N2—C10—C11	76.99 (15)	C25—C26—C27—N5	−0.1 (2)

Symmetry code: (i) −*x*, −*y*+2, −*z*.

### (4,4'-Bipyridyl-κN)bis(N-2-hydroxyethyl-N-isopropyldithiocarbamato-κ2S,S')cadmium(II)–4,4'-bipyridyl (2/1) (II) Hydrogen-bond geometry (Å, º)

Cg1 is the centroid of the Zn/S3/S4/C7 chelate ring.

**Table:**

*D*—H···*A*	*D*—H	H···*A*	*D*···*A*	*D*—H···*A*
O1—H1*O*···N4^ii^	0.83 (2)	1.88 (2)	2.7085 (15)	176 (1)
O2—H2*O*···O1^iii^	0.83 (1)	1.89 (1)	2.7162 (14)	173 (2)
C4—H4···S2^iv^	1.00	2.68	3.5395 (14)	144
C22—H22···O2^v^	0.95	2.40	3.3473 (17)	174
C26—H26···*Cg*1^vi^	0.95	3.00	3.776 (3)	140

Symmetry codes: (ii) −*x*, *y*−1, −*z*+1/2; (iii) *x*+1/2, *y*+1/2, *z*; (iv) *x*+1/2, *y*+3/2, *z*; (v) *x*, −*y*, *z*−1/2; (vi) *x*, *y*+1, *z*.
